# Effect of an Educational Intervention on Health-Related Quality of Life in Patients With Acute Coronary Syndrome: A Non-randomized Clinical Trial

**DOI:** 10.7759/cureus.94795

**Published:** 2025-10-17

**Authors:** Dimitra Anagnostopoulou, John Skoularigis, Foteini Malli, Eleni Tsimitrea, Maria Chatzi, Aikaterini Toska, Maria Saridi, Maria Malliarou, Ioanna V Papathanasiou, Evangelos C Fradelos

**Affiliations:** 1 Department of Nursing, University of Thessaly, Larissa, GRC; 2 Department of Medicine, University of Thessaly, Larissa, GRC; 3 Department of Infectious Diseases, University General Hospital of Larissa, Larissa, GRC

**Keywords:** acute coronary syndrome (acs), health-related quality of life (hrqol), patient education videos, patient-reported outcomes (pros), quasi-experimental study, secondary cardiovascular prevention

## Abstract

Introduction

Acute coronary syndrome (ACS) is a life-threatening condition that requires long-term management after successful treatment in the acute phase. This study aimed to evaluate the effectiveness of a secondary prevention educational program on health-related quality of life (HRQoL) in patients with acute coronary syndrome (ACS) treated with percutaneous coronary angioplasty (PCI).

Methods

This non-randomized controlled, single-blinded, two-parallel-group trial included 122 patients who were recruited from the outpatient cardiology clinic of a university hospital in Greece from July 2022 to September 2024 and allocated equally to the intervention or the control group. Intervention group patients received an individual session, guided by a nurse, that combined the use of a structured educational video, brochure, and individual counseling. Control group patients received standard care and the brochure. Data were collected by completing the health survey (SF-36) questionnaire at baseline before the intervention and after six months of follow-up. The statistical analysis included the chi-square test for nominal data and the paired t-test and mixed design repeated measures analysis of variance (ANOVA) for differences within and between groups at different time points. The statistical significance level was p < 0.05.

Results

No statistically significant differences were found in demographic and clinical scores before intervention between the two groups, confirming equivalence at baseline. Around 111 participants completed the follow-up and were included in the final analysis. Compared to baseline, at the six-month follow-up, both groups recorded statistically significantly improved scores on most dimensions of HRQoL. Using the repeated measures ANOVA test, it was found that the intervention group statistically significantly improved the general health (GH, p=0.023) subscale of HRQoL compared to the control group. Additionally, marginal significance was found in physical functioning (PF, p=0.054) and in role physical (RP, p=0.060).

Conclusions

This study found a statistically significant positive effect of an educational program, based on educational video and individual counseling, on the HRQoL subscale for general health and marginal significance on physical functioning and physical role. These findings highlight a potential low-cost alternative for implementation in clinical practice, especially in healthcare systems and organizations that do not have organized cardiac rehabilitation programs or are affected by limited resources.

## Introduction

Cardiovascular disease (CVD) is the most common cause of mortality and morbidity worldwide [1,2]. Acute coronary syndrome (ACS) is often the first manifestation of CVD [3]. While progress in its treatment in the acute phase has improved survival, secondary prevention is necessary to prevent the recurrence of major cardiac events [3], as these are more frequent in patients with already established coronary artery disease (CAD) [4]. Studies have shown that approximately 75% - 80% of ACS events could be avoided by improving behavioral risk factors [1,5], while adapting to a healthy diet and exercise pattern and avoiding smoking, alcohol abuse, and weight reduction, offering additional benefits beyond medical interventions [6]. The goals of secondary prevention, through pharmacotherapy, medical monitoring, risk factor management, and lifestyle optimization, are to reduce premature morbidity and mortality, reduce rising healthcare costs [3,7], avoid disability, and improve quality of life, and therefore, should be initiated as soon as possible after ACS [3,8,9].

Health-related quality of life (HRQoL), which reflects an individual's subjective perception of their health status as a result of their disease, is considered a crucial clinical outcome measure in research on patients with CAD and a key indicator of treatment effectiveness. As an outcome measure, it is valuable, as there is a discrepancy between self-reported health status and physician-reported health status [10]. Furthermore, recent studies have shown that HRQoL has predictive value for long-term mortality and ACS in patients with CAD [10,11,12].

To improve HRQoL and prevent further events, international cardiology societies recommend that patients be referred to comprehensive cardiac rehabilitation programs after an ACS episode [3,8,13]. These programs include regular medical monitoring, a predetermined number of regular supervised exercise sessions, education, and psychosocial support. However, globally, their availability is inadequate, and, even more so, patient participation and program completion are limited [14]. Thus, it is common practice to develop and implement patient education programs with the aim of changing their health behaviors or improving their health status. Education is essential, as a large proportion of patients have a low level of health literacy, which negatively affects their ability to understand, search for, evaluate, and apply health information for their self-care [3]. Furthermore, the provision of education in recent years has been facilitated by the use of digital media, such as videos, virtual reality, or mobile applications.

The effect of these educational programs on HRQoL has been studied in numerous primary studies, but with inconsistent results. Furthermore, these studies have used many different HRQoL assessment measures, such as general scale instruments, like the SF‐36, or disease‐specific HRQoL instruments, like the MacNew Heart Disease Quality of Life Questionnaire. Therefore, their comparison, as well as conducting meta-analysis, is difficult [15,16]. Systematic reviews that have studied the effect of education on HRQoL have reported evidence of improvement in some studies, all in favor of the intervention groups, but either with small effect sizes [17] or in HRQoL subscales, without consistent results across domains [15,16]. Furthermore, the effect on HRQoL of short-term educational programs that integrate digital media into the educational process has not been adequately documented. Finally, the effect of such studies has not been evaluated in the Greek population.

The primary objective of this study was to evaluate the impact of an educational program, based on educational video and individual counseling, on HRQoL in patients with ACS treated with percutaneous coronary angioplasty (PCI) six months after the intervention. The findings of this study are expected to contribute to a deeper understanding of the impact of this type of intervention and to the development of more effective care strategies in Greece.

## Materials and methods

Study settings and participant recruitment

This study was a non-randomized controlled, single-blind, two-parallel-group clinical trial conducted at the Outpatient Cardiology Clinic of the University General Hospital of Larissa from July 2022 to March 2025. The study included patients aged 18-75 years with ACS who received PCI treatment. Participants were informed that the experimental study included individual education for secondary prevention and monitoring of treatment adherence. After their informed consent, they were equally assigned to the intervention or control group, matched for demographic and clinical characteristics. However, they were blinded to educational program allocation. Their demographic and clinical data and the HRQoL questionnaire were completed before the intervention and six months after. The study was conducted by a trained nurse-researcher, who, due to the nature of the intervention, could not be blinded. Doctors and nurses involved in the standard treatment and regular outpatient follow-up of the patients, as well as the statistician who performed the final data processing, were blinded to the allocation.

Sample size

The sample size was calculated with the statistical program G*Power 3.1.9.4 (Heinrich-Heine-Universität Düsseldorf, Düsseldorf, Germany). Given an effect size of =0.25, power (1-β err prob)=0.85 and a statistical significance level of p < 0.05, for the ANOVA statistical test: Repeated measures, between factors between two groups for two consecutive measurements, was calculated that 55 patients were needed, per group, plus a 10-12% increase due to possible losses to follow-up. Thus, a total of 122 patients were included in the study, 61 in the intervention group and 61 in the control group.

Eligibility criteria

The participants were recruited from patients hospitalized in the cardiology clinic with a diagnosis of ACS and had been treated with PCI. The study's inclusion criteria required participants to be over 18 years old, able to speak and write Greek, and provide written consent. The exclusion criteria included individuals over 75 years old, those with a history of psychiatric or cognitive disorders, motor, visual, or auditory disabilities, substance abuse issues, and serious comorbidities such as heart failure or cancer that would significantly reduce life expectancy.

Measuring instruments

Demographic and Clinical Information Questionnaires

Socio-demographic and clinical characteristics were collected using a questionnaire tailored to the study’s needs (Appendix 1). Demographic data included age, gender, marital status, place of residence, education, employment, and insurance status of the participants, while clinical data included type of ACS, left ventricular ejection fraction (LVEF) before discharge, type of procedure (primary PCI, PCI), family and personal history of CAD, comorbidities, risk factors, laboratory test results, and somatometric data such as weight, height, and waist circumference. Overweight was defined as a body mass index (BMI) ≥25 to <30 kg/m², and obesity as a BMI ≥30 kg/m² [18].

HRQoL Questionnaire

HRQoL was assessed using the self-report SF-36 questionnaire, which records a subjective measurement of eight dimensions of HRQoL: physical functioning, physical role, physical pain, general health, vitality, social functioning, emotional role, and mental health. These subscales form the physical function summary scale (PCS) and the mental function summary scale (MCS). The total score ranges from 0 to 100, with higher scores indicating better HRQOL. This questionnaire has been translated and standardized in the Greek population and is free to use [19,20]. All dimensions, as well as summary scales, were used as outcome measures in this study.

Self-Report on Current Behavior Questionnaire

Data was collected using a self-report questionnaire appropriately designed for the needs of the study (Appendix 2). It included recording scales for diet, smoking, and physical activity. The smoking status subscale is based on WHO (2011) guidelines [21], such as smoking initiation, duration, and smoking status in the last month, with a description of the type of tobacco product and number of cigarettes per day. The physical activity subscale is based on the proposed classification of the intensity of different types of physical activity during a week by the European Society of Cardiology (ESC) (2021) [8]. The diet subscale includes a three-day food record from the last week.

Educational material

For the study’s needs, a 22-minute educational video and an 18-page information brochure were created. Their content was selected considering the current literature and in accordance with the ESC (2021) guidelines for the long-term management of ACS [8]. The final content was approved by cardiology professors of the Department of Medicine and professors of the Department of Nursing of the University of Thessaly.

The educational content of the video entitled “Treatment goals and recommendations for secondary prevention of CAD after angioplasty” was created in the Greek language with visual material, mainly in the form of animation. Its aim was to present a concise and comprehensive range of topics related to disease and its manifestations and to highlight the need for lifelong self-management through the appropriate adaptation of basic health behaviors. It included topics related to normal cardiovascular function, the process of development of atherosclerotic plaques, the clinical manifestations of ACS, and the warning symptoms so that they can recognize them immediately and respond appropriately. It also included the procedure of performing angioplasty with an endovascular stent and the goals of secondary prevention after it, such as adherence with medication, diet, regular exercise, abstinence from smoking and alcohol abuse, and control of hypertension, weight, and blood sugar, as well as stress management.

The information brochure "Therapeutic goals and recommendations for the secondary prevention of coronary heart disease" contained similar content to that of the video, in a more concise description using simple language and including images where necessary.

Intervention procedure

The intervention was carried out one month after hospital discharge at the outpatient Cardiology Clinic, approximately one and a half hours earlier than each participant's scheduled appointment with their cardiologist. Initially, a clinical examination was performed by the cardiology clinic nurses, and blood pressure (digital measurement with Philips EarlyVue VS30 Monitor, Koninklijke Philips N.V., Amsterdam, Netherlands), weight, and height (measured with Wunder Sa.Bi. s.r.l., Milan, Italy) were measured. The appointment with the study nurse had the same first step for all participants. In most cases, the participants' companions also participated in the session. After signing the informed consent form, participants completed demographic information, self-report health behavior questionnaires, and the SF-36 questionnaire. The researcher completed the questionnaire with clinical data from the clinic's medical records and the clinical examination, and was available to help when participants had difficulty reading or understanding the questions.

In the intervention group, the educational session, lasting approximately 55-70 minutes, began with watching the study video. If necessary, the video was paused to explain medical terms and any points that were not sufficiently understood. Afterwards, a discussion and counseling on the management of individual risk factors and a summary of the goals for secondary prevention followed. To confirm the patient's understanding of the individual prevention goals, the "teach back" method was used [8]. In addition, the study information brochure was given, flipped through, and explained to each participant, with the recommendation to study it at home and refer to it when they needed to remember a specific topic.

The control group received brief verbal information about the general goals of secondary prevention, and, to conceal group allocation from patients and clinic staff and to be consistent with our commitment to education, they also received the study information brochure. Therefore, we consider this minimal education provided to the control group to constitute standard care. Participants in both groups received standard care from the doctors and nurses of the Cardiology Clinic.

Follow-up data collection

Follow-up data collection for both groups was conducted in person and in the same manner, six months after the intervention in the cardiology outpatient clinic, before the participants' regular appointment with their cardiologist. If a participant had not scheduled an appointment, a reminder call was made.

Statistical analysis

Descriptive and inferential statistics were performed with IBM SPSS Statistics for Windows, Version 23 (Released 2015; IBM Corp., Armonk, New York, United States). Chi-square test was used to compare nominal data, while paired t-test and mixed design repeated measures ANOVA were used to test differences within and between groups at different time points. Statistical significance was set at p < 0.05.

Ethical considerations

The present study was approved by the Scientific Council of the University General Hospital of Larissa (Protocol No. 3675/28-01-2022) and by the Ethics and Deontology Committee of the Department of Nursing of the University of Thessaly (Protocol No. 76/17-01-2022). All participants were informed of their rights to refuse or discontinue participation in the study according to the ethical standards of the Declaration of Helsinki (1989) of the World Medical Association and gave written, informed consent before their enrollment. Data collection guaranteed anonymity and confidentiality.

## Results

Recruitment, attrition, and intervention engagement

**Figure 1:**
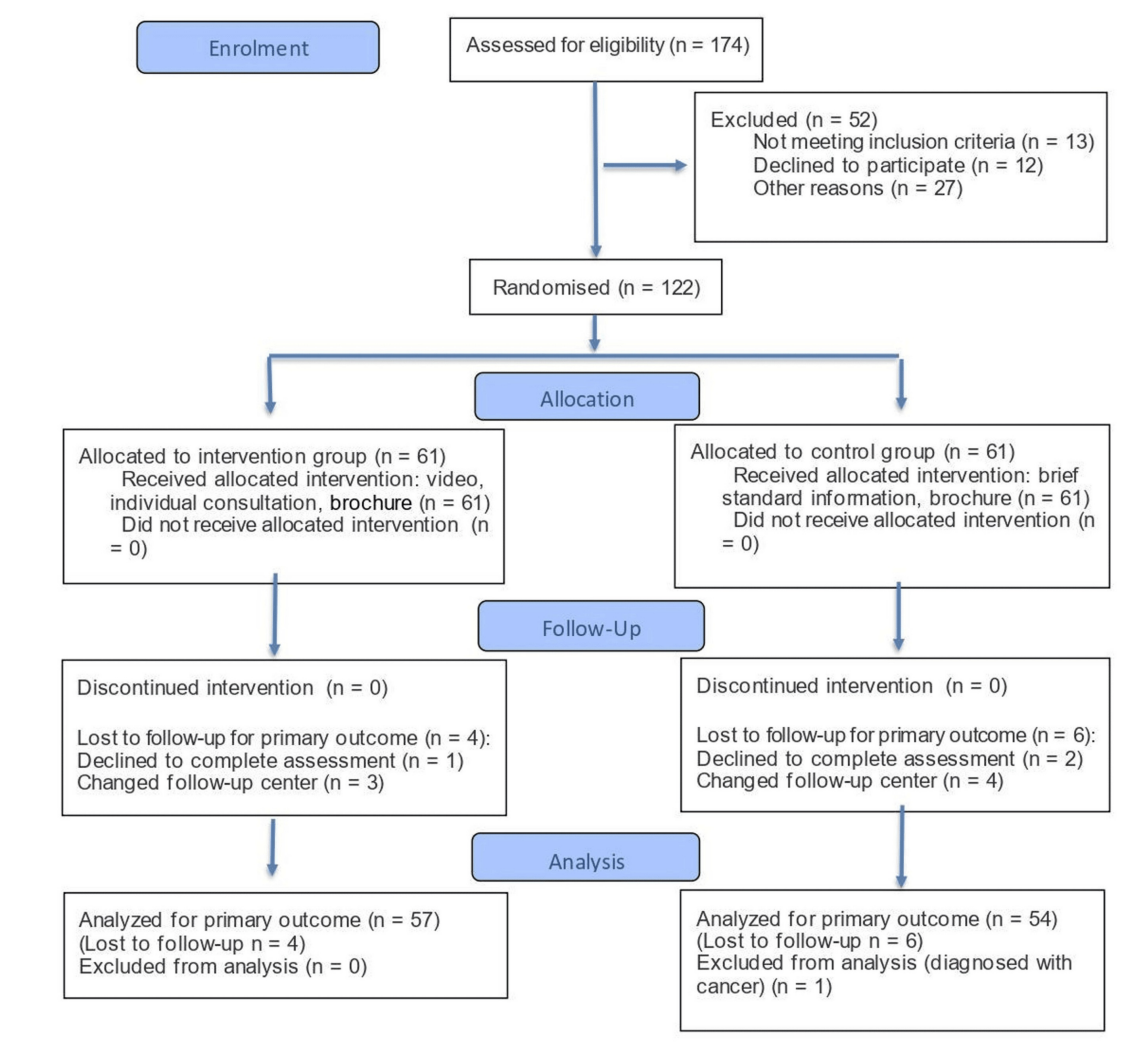
Study flow diagram

A total of 122 patients met the inclusion criteria and were recruited, 61 per study group. Of these, 111 completed the study with data collection at six-month endpoints and were included in the final analysis for the primary outcome: 57 from the intervention group and 54 from the control group. The study flow diagram, according to Consolidated Standards of Reporting Trials (CONSORT) (2025) guidelines [22], is presented in Figure 1.

Participants characteristics

Around 122 patients with ACS were included, with a mean age of 56.43 years (range: 32 - 75). The patient profile was that of a middle-aged male, a high school graduate, married, and employed with ST-segment elevation myocardial infarction (STEMI). The most common risk factors were hypercholesterolemia (n=97, 79.5%), followed by smoking at the time of the event (n=86, 70.5%), hypertension (n=58, 47.5%), obesity (n=45, 36.9%), and diabetes mellitus (n=19, 15.6%), while regular physical activity was reported by 36 (29.5%) participants. The basic demographic and clinical parameters per group are summarized in Tables 1, 2.

**Table 1:** Age and BMI of participants *Student's t-test was used for intergroup comparisons, with a significance level of p<0.05. std. error: standard error of the mean; BMI: body mass index

Varaibles	Control group (n=61)	Intervention group (n=61)	P*
Continuous variables	Mean±	Mean±	
	Std. Error	Std. Error	
Age	56.80±1.03	56.05±1.22	0.639
ΒΜΙ	29.39±9.87	28.13±4.78	0.380

**Table 2:** Study participants' baseline demographic and nosological characteristics (n=122) Values ​​are expressed numerically and as frequency (percentage), n = number of patients. * chi-square test was used for intergroup comparisons, with a significance level of p<0.05. ** including respiratory diseases, endocrinological diseases, and urogenital diseases. *** patient-reported physical activity of at least 150 minutes per week of low-to-moderate intensity or 75 minutes of vigorous intensity, or an equivalent combination. df: degrees of freedom; STEMI: ST-segment elevation myocardial infarction; NSTEMI: non-ST-segment elevation myocardial infarction; UA: unstable angina; LVEF: left ventricular ejection fraction; ACS: acute coronary syndrome; CAD: coronary artery disease

Variables		Control group (n=61)		Intervention group (n=61)		df	Effect size	P*
Discrete variables		N	%	N	%			
Gender	Men	55	90.2	55	90.2	1	0.006	1
	Women	6	9.8	6	9.8			
Family status	Single	10	16.4	8	13.1	2	0.240	0.260
	Married/cohabitant	44	72.1	52	85.2			
	Divorced/widowed	7	11.5	1	1.6			
Educational level	Elementary	10	16.4	6	9.8	4	0.149	0.955
	Junior high school	10	16.4	12	19.7			
	High school	21	34.4	21	34.4			
	College	8	13.1	8	13.1			
	University	12	19.7	14	23.0			
Profession	Employee	18	29.5	17	27.9	5	0.195	0.994
	Civil servant	7	11.5	8	13.1			
	Freelancer	10	16.4	10	16.4			
	Farmer	6	9.8	5	8.2			
	Unemployed	6	9.8	4	6.6			
	Pensioner	14	23.0	17	27.9			
Children (no)	None	16	26.2	12	19.7	3	0.207	0.067
	1	15	24.6	11	18.0			
	2	21	34.4	31	50.8			
	≥3	9	14.8	7	11.5			
Living alone	Yes	10	16.4	8	13.1	1	0.039	0.718
	No	51	83.6	53	86.9			
Cardiovascular risk factors	Hypertension	26	41.9	32	53.3	1	0.080	0.357
	Hypercholesterolemia	48	77.4	49	81.7	1	0.090	0.684
	Diabetes	12	21.0	7	11.7	1	0.088	0.331
	Obesity	21	33.9	24	40.0	1	0.044	0.617
	Family history of CAD	13	21.0	13	21.7	1	0.078	1.000
	Personal history of CAD	6	9.7	8	13.3	1	0.040	0.652
	Comorbidities**	16	25.8	15	25.0	1	0.071	0.364
	Physical activity***	13	21.3	23	37.7	1	0.196	0.140
Cardiovascular risk factors-smoking	Current smokers	46	75.4	40	65.6	2	0.119	0.518
	Non-smokers	4	6.6	9	14.7			
	Former smokers	11	8.0	12	19.7			
ACS severity	STEMI	35	57.4	39	63.9	2	0.068	0.757
	N-STEMI	18	29.5	17	27.9			
	UA	8	13.1	5	8.2			
LVEF	Low LVEF	8	12.9	11	18.3	1	0.043	0.693

The two groups shared similar baseline characteristics, and no statistical differences were traced between them, confirming baseline equivalence.

Effects on clinical outcomes

Both groups had a significant effect on health quality dimensions, as measured with SF-36 (Table 3). All parameters showed improvement, apart from mental health in the control group (values were slightly lower after the standard intervention). Of note, the differences were statistically very significant in almost all dimensions in the experimental group before and after the intervention, apart from bodily pain (p=0.077) and mental health (marginal significance, p=0.05). On the contrary, in the control group, no statistical difference was observed in the domains of general health, vitality, and mental health. When the effect of the group on the outcome was investigated, the experimental intervention had a significant effect on general health, the difference (after-before) being equal to 8.37 vs. 2.25 in the control group. Additionally, marginal significance was found in physical functioning (p=0.054) and in role physical (p=0.060). There were no adverse events associated with this study.

**Table 3:** The effect of interventions on health quality dimensions depending on groups *after-before (within groups, paired-t test), ** after-before (between groups, mixed design repeated measures ANOVA), statistical significance was set at p<0.05.

Variables	Control (n=54)				Intervention (n=57)				Between groups
	Before	After			Before	After			
	Mean±	Mean±	Diff*.,	p*	Mean±	Mean±	Diff*.,	p*	p**
	Std. Error	Std. Error	(95% C.I.)		Std. Error	Std. Error	(95% C.I.)		
1. Physical Functioning (PF)	75.19± 2.52	83.11± 2.37	7.92 (3.15- 12.69)	0.002	80.53± 1.99	94.21± 1.02	13.68 (10.09- 17.28)	<0.001	
									0.054
2. Role Physical (RP)	26.89± 4.14	66.04± 5.64	39.1 (27.25- 51.05)	<0.001	35.09± 4.80	88.60± 3.13	53.51 (44.01- 63.01)	<0.001	0.060
3. Bodily Pain (BP)	83.36± 3.28	91.38± 2.46	8.02 (2.12- 13.91)	0.009	89.35± 2.54	93.67± 2.14	4.32 ((-0.49)- 9.12)	0.077	0.328
4 General Health (GH)	70.85± 1.99	73.09± 2.24	2.25 (-1.90- 6.39)	0.282	73.56± 2.29	81.93± 2.02	8.37 (4.95- 11.78)	<0.001	0.023
5. Vitality (VT)	66.04± 2.64	69.43± 2.25	3.40 (-1.03- 7.82)	0.129	71.75± 2.51	78.77± 1.81	7.02 (2.94- 11.10)	0.001	0.229
6. Social Functioning (SF)	67.22± 4.51	87.74± 2.62	20.52 (11.38- 29.66)	<0.001	64.69± 4.19	89.04± 2.52	24.34 (15.95- 32.74)	<0.001	0.537
7. Role Emotional (RE)	58.49± 5.31	79.87± 4.15	21.38 (8.13- 34.64)	0.002	59.65± 4.92	94.15± 2.06	34.50 (24.48- 44.53)	<0.001	0.113
8. Mental Health (MH)	71.47± 1.96	70.26± 2.24	-1.21 ((-5.31)- 2.90)	0.558	75.93± 2.16	79.37± 1.75	3.44 (0.00- 6.87)	0.050	0.083
Α. Physical Quality of Life Score (PCS-12)	45.38± 1.00	51.16± 1.08	2.76 (0.09- 5.43)	0.043	47.84± 0.85	55.14± 0.63	7.30 (5.70- 8.90)	<0.001	0.239
Β. Mental Quality of Life Score (MCS-12)	47.29± 1.17	50.05± 1.06	7.92 (3.15- 12.69)	0.002	47.89± 1.37	53.49± 0.88	5.61 (3.27- 7.94)	<0.001	0.109

## Discussion

This study was conducted to evaluate the effectiveness of a face-to-face educational program that combined the use of structured educational video with personalized information and counseling on HRQoL in ACS patients. Given that there was a limited number of similar published studies with inconsistent results, especially in Greece, where, to the best of our knowledge, this was the first quasi-experimental study with two parallel groups that evaluated the effect of educational programs in patients with ACS, the present study addressed a critical gap in the literature.

The affected initial HRQoL measurement probably reflects the impact of ACS on physical and mental health, as the measurement was made one month after the event, when the patients were still in the recovery phase, while the improvement in the following six months in both groups indicates the expected improvement from reperfusion interventions, according to the literature [9,23], but also the effective existing standard care by cardiologists for the long-term management of the disease. To some extent, the improvement observed in the control group may have been influenced by the brief verbal information and printed material provided to all study participants. These hypotheses are also supported by the findings of two cross-sectional studies in Greece. The first found that patients with CAD had moderate levels of quality of life at baseline before PCI, which gradually increased to statistically significant levels in all SF-36 subscales at six and 12 months of follow-up [23]. The second study found that patients with ischemic heart disease of the wider region had lower mean values ​​of the PCS and MCS scales of the SF-36 than those found in our study for both groups at the six-month follow-up endpoint [24].

Nevertheless, in the experimental group, higher improvements were found in almost all dimensions. Of particular importance is the improvement observed in the mental health of participants in the intervention group, in contrast to the slight decreasing trend observed in the control group. These findings indicate that despite the high level of standard care, there is potential to improve the quality of life of patients after ACS, especially in mental health, with appropriate educational interventions. Furthermore, the study by Tsoulou et al. (2023) found that improvement in the general and mental health dimensions was significantly associated with the frequency of physical activity [23]. We hypothesize that improvements in the same dimensions in our experimental group likely reflect the positive effect of education on lifestyle modification and self-management behavior, leading to improved mental health. This is aligned with the findings of two meta-analyses, which found that patient education programs for secondary prevention improve adherence to lifestyle modification [25] and, furthermore, mental health in CAD patients, even if they did not include targeted psychological interventions [26].

While we found no statistical difference in all dimensions of HRQoL at follow-up between the intervention and comparison groups, these findings align with systematic reviews reporting HRQoL outcomes [15,16]. A review found that of the fifteen studies addressing this outcome, some studies reported positive effects of education on subscales but not on overall HRQoL, and the only study that demonstrated improvement in almost all SF-36 domain scores in the intervention group and no difference in the control group did not perform a direct statistical comparison between the intervention and control groups [16]. Similarly, regarding studies that have integrated digital media into the educational process, some have reported a positive effect on HRQoL outcomes. A study using the MacNew scale reported significant differences in the physical and social domains in favor of the intervention group at three months follow-up [27]. Ma et al.'s study, using the SF-12, measured the PCS and MCS subscales at three, six, nine, and 12 months and found no significant effect at three and six months but found significant improvements at nine and 12 months [28]. Finally, a study by Sorlie et al., using a modified SF-36 subscale, performed repeated measures and found a significant effect on subjective health at all time points up to two years of follow-up [29], indicating maintenance of the positive effect on this outcome over time. In our study, we only performed one measurement at the six-month endpoint and found a significant effect on general health and marginal significance on physical functioning and physical role.

Considering the results of relevant studies, we assessed that our educational program, whilst of brief duration, showed comparative effectiveness in improving patients’ HRQoL aspects. We assume that the integrated video with structured and comprehensive content and the individual consultation contributed to the observed effect.

Strengths, limitations, and future suggestions

The present study planned to implement the educational session consistently, without deviations, and this was the main strength of the study, as it is easily reproducible. The implementation of the intervention in an outpatient clinic at the same time as a regular appointment with a cardiologist and the use of video to provide educational content in the presence, guidance, and advice of the nurse ensured the fidelity of the educational session. The second advantage was that its design ensured the optimal use of time and resources for both the provider and the participants.

However, this study had several limitations that should be considered alongside the findings. First, this study was not prospectively registered in a repository before the first participant was enrolled, and although the main objectives of the study remained unchanged according to the protocol, this is a limitation for publication bias. Second, participants were recruited from a single hospital. Although it was a tertiary university, a referral center of a large region, the characteristics of the sample may differ from broader populations. Third, although the two groups did not differ in demographic and clinical characteristics, the lack of randomization is likely to have introduced selection bias. Fourth, the sample size was calculated using power analysis for a moderate effect size. Future studies with larger sample sizes may be able to detect smaller differences. Five, the comparison group received printed materials in addition to standard care, the influence of which was difficult to quantify and may have attenuated the differences between the two groups. Finally, the study measured the effect of education only once at the six-month post-intervention endpoint. Although this follow-up period is appropriate for assessing the maintenance of behavior change over time [26], future studies should incorporate more measurements and a long-term assessment of the effects of education on HRQoL.

## Conclusions

This study found a statistically significant positive effect of an educational program on the HRQoL subscale for general health and marginal significance on physical functioning and physical role in patients after ACS and PCI at six months of follow-up. These findings demonstrate that even short programs that have structured content and combine digital educational tools and face-to-face consultations can be effective in improving health outcomes. Therefore, this finding highlights a potential low-cost alternative that can be easily implemented in clinical practice, especially for healthcare systems and organizations that do not have organized cardiac rehabilitation programs or are affected by a lack of human and financial resources.

Nevertheless, while the study offers initial information, it does not provide definitive evidence for its long-term impact and broad policy implementation. Future studies should incorporate more time-point measurements, as a long-term evaluation could provide deeper insights into the evolution of the observed effects over time.

## Figures and Tables

**Figure 2:**
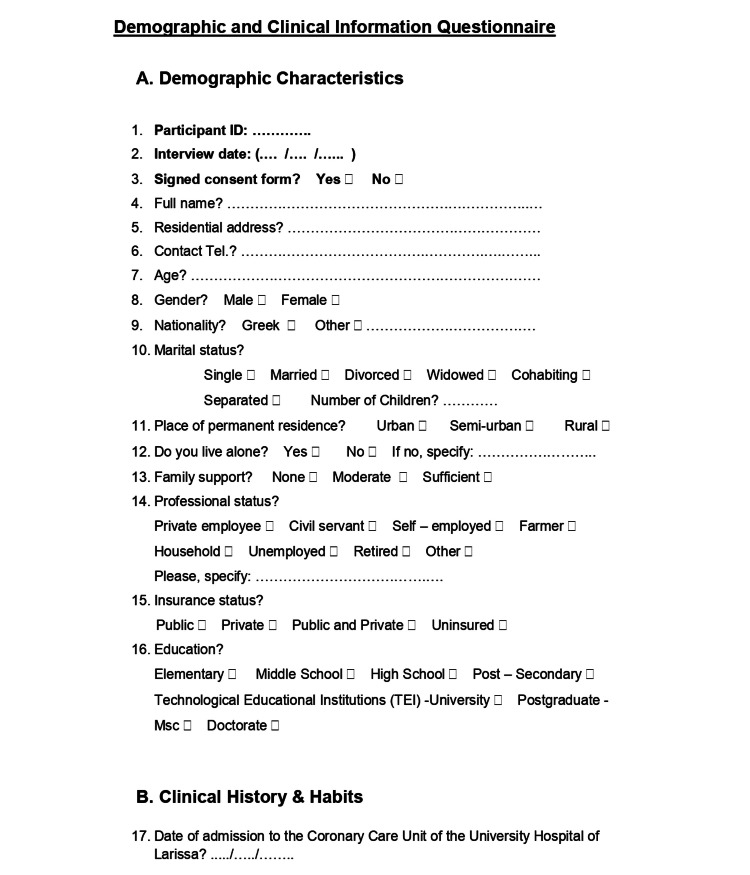
Demographic and clinical questionnaire (part 1)

**Figure 3:**
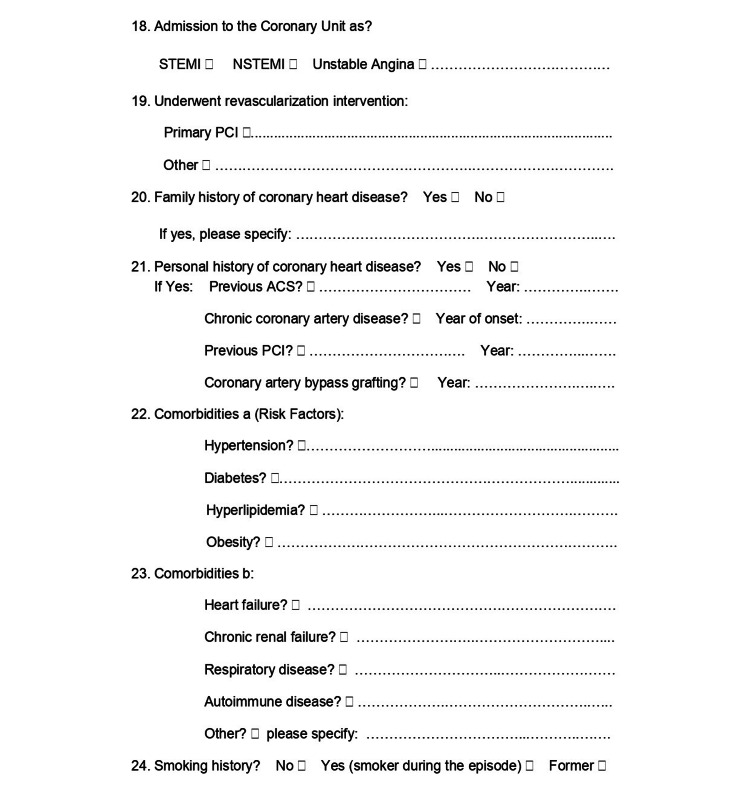
Demographic and clinical questionnaire (part 2)

**Figure 4:**
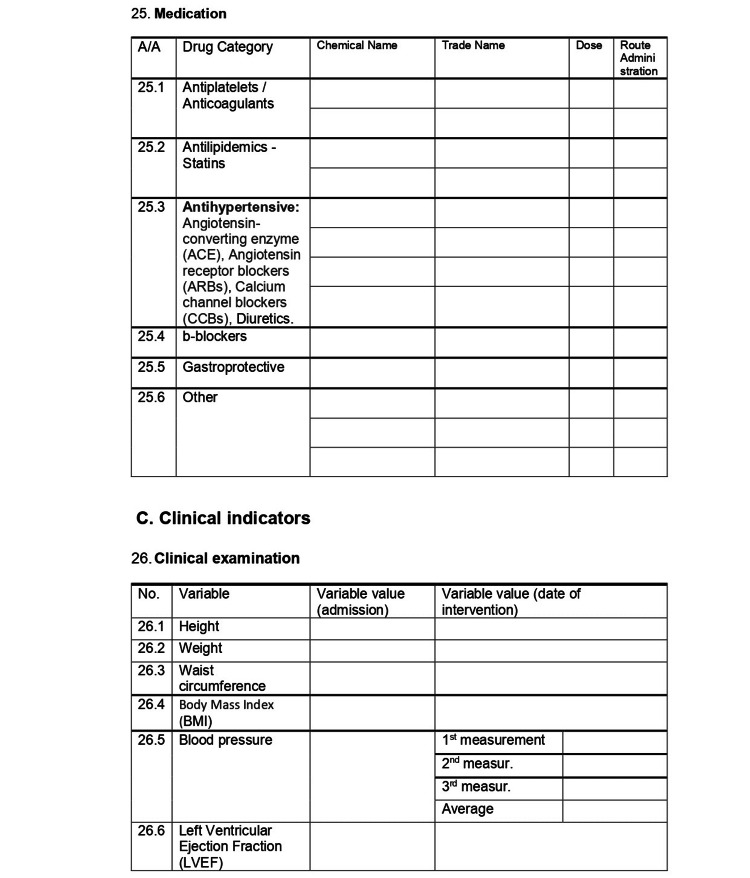
Demographic and clinical questionnaire (part 3)

**Figure 5:**
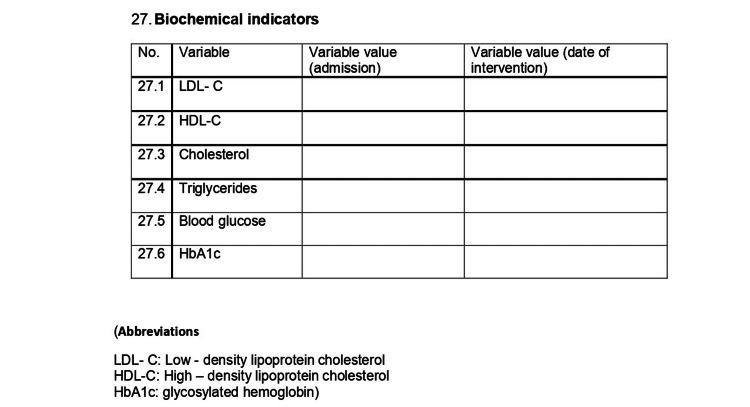
Demographic and clinical questionnaire (part 4)

**Figure 6:**
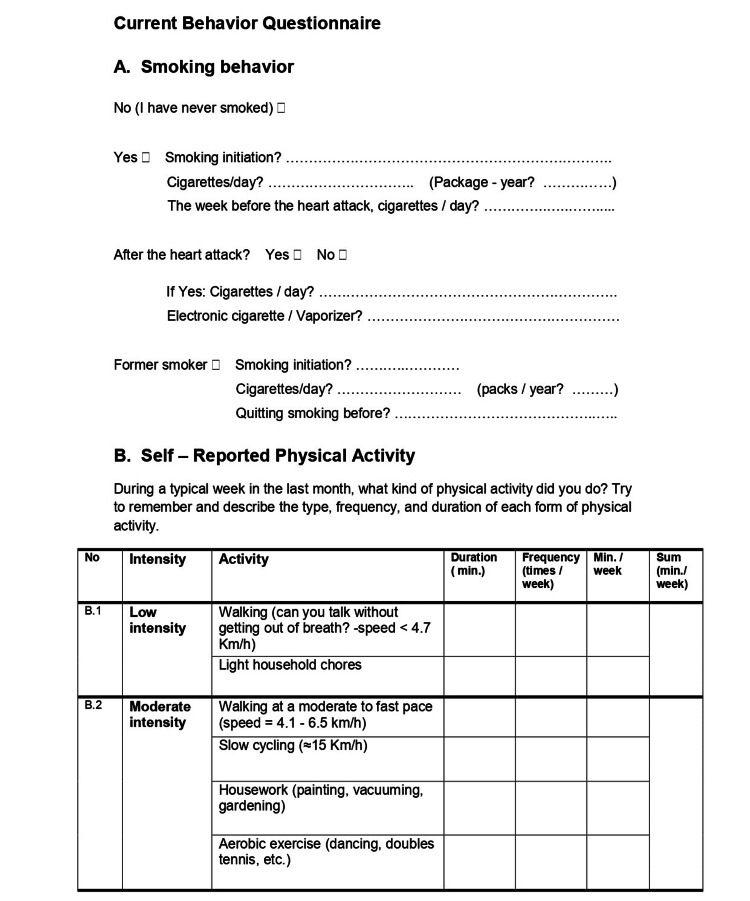
Current behavior questionnaire (part 1)

**Figure 7:**
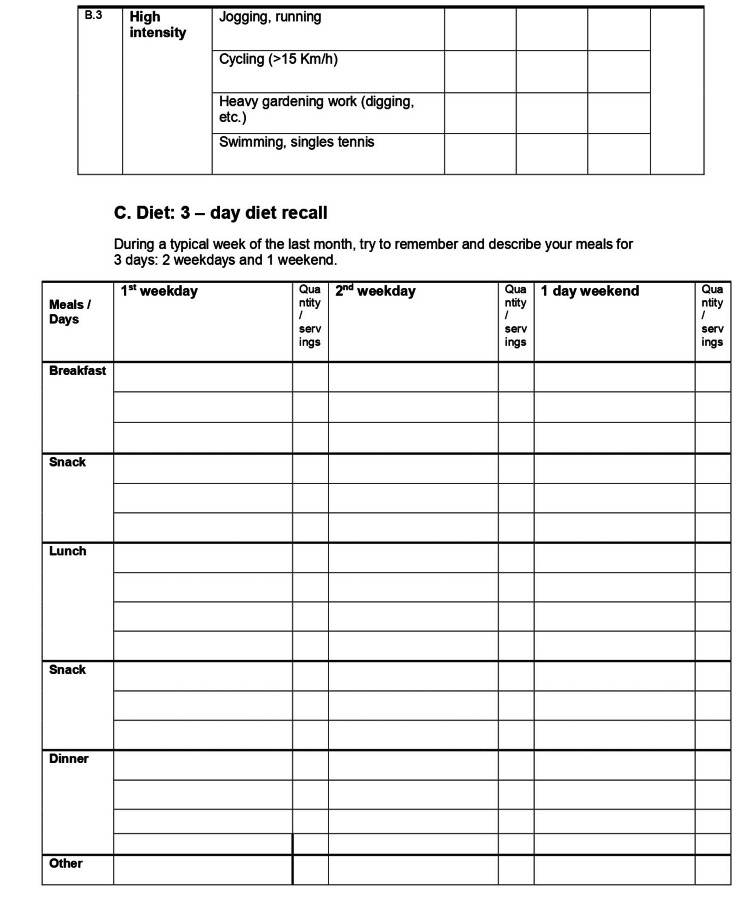
Current behavior questionnaire (part 2)

**Figure 8:**
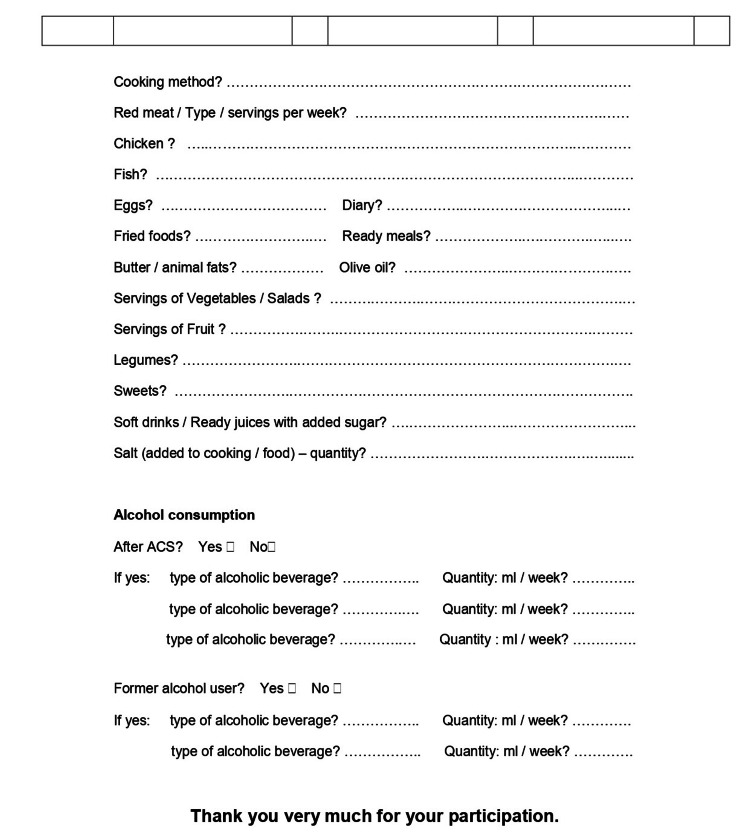
Current behavior questionnaire (part 3)

## Appendices

Appendix 1

Appendix 2
